# A prediction nomogram for the 3-year risk of incident diabetes among Chinese adults

**DOI:** 10.1038/s41598-020-78716-1

**Published:** 2020-12-10

**Authors:** Yang Wu, Haofei Hu, Jinlin Cai, Runtian Chen, Xin Zuo, Heng Cheng, Dewen Yan

**Affiliations:** 1 Department of Endocrinology, The First Affiliated Hospital of Shenzhen University, No.3002 Sungang Road, Futian District, Shenzhen, 518035 Guangdong Province China; 2 Department of Nephrology, The First Affiliated Hospital of Shenzhen University, Shenzhen, 518035 Guangdong Province China; 3 Department of Endocrinology, Shenzhen Second People’s Hospital, Shenzhen, 518035 Guangdong Province China; 4 Department of Nephrology, Shenzhen Second People’s Hospital, Shenzhen, 518035 Guangdong Province China; 5 Shenzhen University Health Science Center, Shenzhen, 518071 Guangdong Province China; 6 Shantou University Medical College, Shantou, 515000 Guangdong Province China; 7 Department of Endocrinology, Shenzhen Third People’s Hospital, Shenzhen, 518116 Guangdong Province China

**Keywords:** Diseases, Endocrinology, Health care, Medical research, Risk factors

## Abstract

Identifying individuals at high risk for incident diabetes could help achieve targeted delivery of interventional programs. We aimed to develop a personalized diabetes prediction nomogram for the 3-year risk of diabetes among Chinese adults. This retrospective cohort study was among 32,312 participants without diabetes at baseline. All participants were randomly stratified into training cohort (n = 16,219) and validation cohort (n = 16,093). The least absolute shrinkage and selection operator model was used to construct a nomogram and draw a formula for diabetes probability. 500 bootstraps performed the receiver operating characteristic (ROC) curve and decision curve analysis resamples to assess the nomogram's determination and clinical use, respectively. 155 and 141 participants developed diabetes in the training and validation cohort, respectively. The area under curve (AUC) of the nomogram was 0.9125 (95% CI, 0.8887–0.9364) and 0.9030 (95% CI, 0.8747–0.9313) for the training and validation cohort, respectively. We used 12,545 Japanese participants for external validation, its AUC was 0.8488 (95% CI, 0.8126–0.8850). The internal and external validation showed our nomogram had excellent prediction performance. In conclusion, we developed and validated a personalized prediction nomogram for 3-year risk of incident diabetes among Chinese adults, identifying individuals at high risk of developing diabetes.

## Introduction

Diabetes mellitus has become a significant public health issue all over the world. Due to the aging population and unhealthy lifestyles, the prevalence of diabetes worldwide is rapidly increasing. It was estimated that there were 451 million (age 18–99 years) people with diabetes in 2017, and the number was expected to increase to 693 million by 2045^1^. The global burden of disease study identified that diabetes resulted in 1.37 million deaths in 2017^2^. Due to its high morbidity, disability and mortality, diabetes has a major impact on society, economy, and development worldwide. China has the world’s most enormous numbers of diabetic patients, reaching up to 109.6 million^3^. However, more than half of Chinese adults with diabetes were undiagnosed^4^.

As a debilitating chronic epidemic, early identification and diagnosis, early treatment is an essential part of diabetes prevention and health care. The central component of diabetes preventive strategies is to identify individuals at high risk for incident diabetes^5^. Studies demonstrated that lifestyle modification and pharmacological intervention could prevent or delay the occurrence of diabetes^6,7^. Moreover, for newly diagnosed diabetic patients, intensive lifestyle intervention, metabolic surgery and early short-term intensive insulin therapy can induce long-term glycemic remission without further antidiabetic medication^8–12^. Several studies have shown that early diagnosis and timely treatment can delay the progression of diabetes, delay or even prevent the occurrence of diabetes complications^13–15^. Therefore, it is crucial to find a feasible and accurate screening tool to identify those with undiagnosed diabetes or at high risk of the onset of diabetes, which will be beneficial for the effective implementation of diabetes prevention programs.

Risk prediction models have considerable potential to contribute to the decision-making process regarding the clinical management of a patient^16^. The models can screen individuals to identify at an increased risk of having an undiagnosed condition, for which diagnosis management and treatment can be initiated and ultimately improve patient outcomes. A variety of risk prediction models for screening diabetes have been established, mainly applied to western populations^17–23^. These predictive models may not apply to the Chinese population due to the differences in diet, lifestyle, social environment, and genetic predisposition. The least absolute shrinkage and selection operator (LASSO) method is suitable for reducing high-dimensional data and is performed to select the most useful prediction candidates^24,25^. Nomogram is an intuitive graphical prediction model that can provide accurate and individualized risk predictions for each individual. However, there were only a limited number of prediction nomogram for risk of diabetes in China^26–28^. And the existing diabetes risk prediction models incorporate many variables, which are not convenient to apply. Besides, they are mainly single-center studies, and none of them has conduct external validation. Therefore, we aimed to introduce the LASSO method to select the least and optimal variables to predict the 3-year risk of incident diabetes. Furthermore, we sought to develop and validate a personalized diabetes prediction nomogram by more cost-effective and readily available parameters in a large cohort of Chinese adults across 32 sites and 11 cities to help clinicians accurately identify individuals at high risk for diabetes and guide them in timely diabetes screening.

## Materials and methods

### Study design and participants

The data was obtained from a public, non-profit computerized database established by the Rich Healthcare Group in China, namely, the ‘DATADRYAD’ database (www.Datadryad.org). We downloaded the raw data shared by Chen et al.^29^ from: Association of body mass index and age with incident diabetes in Chinese adults: a population-based cohort study. Dryad Digital Repository. http://dx.doi.org/10.1136/bmjopen-2018-021768. And the raw data is available publicly for use. The original study enrolled 685,277 participants ≥ 20 years old with at least two routine health checks from 2010 to 2016 across 32 sites and 11 cities in China (Shanghai, Beijing, Nanjing, Suzhou, Shenzhen, Changzhou, Chengdu, Guangzhou, Hefei, Wuhan, Nantong).

Variables were extracted as follows: age, gender, smoking status, drinking status, family history of diabetes, body mass index (BMI), systolic blood pressure (SBP), diastolic blood pressure (DBP), fasting plasma glucose (FPG), total cholesterol (TC), triglyceride(TG), low-density lipoprotein cholesterol(LDL-C), high-density lipoprotein cholesterol (HDL-C), serum urea nitrogen(BUN), serum creatinine(Scr), alanine aminotransferase(ALT) at baseline, years of follow up, a censor of diabetes at follow up.

**Figure 1:**
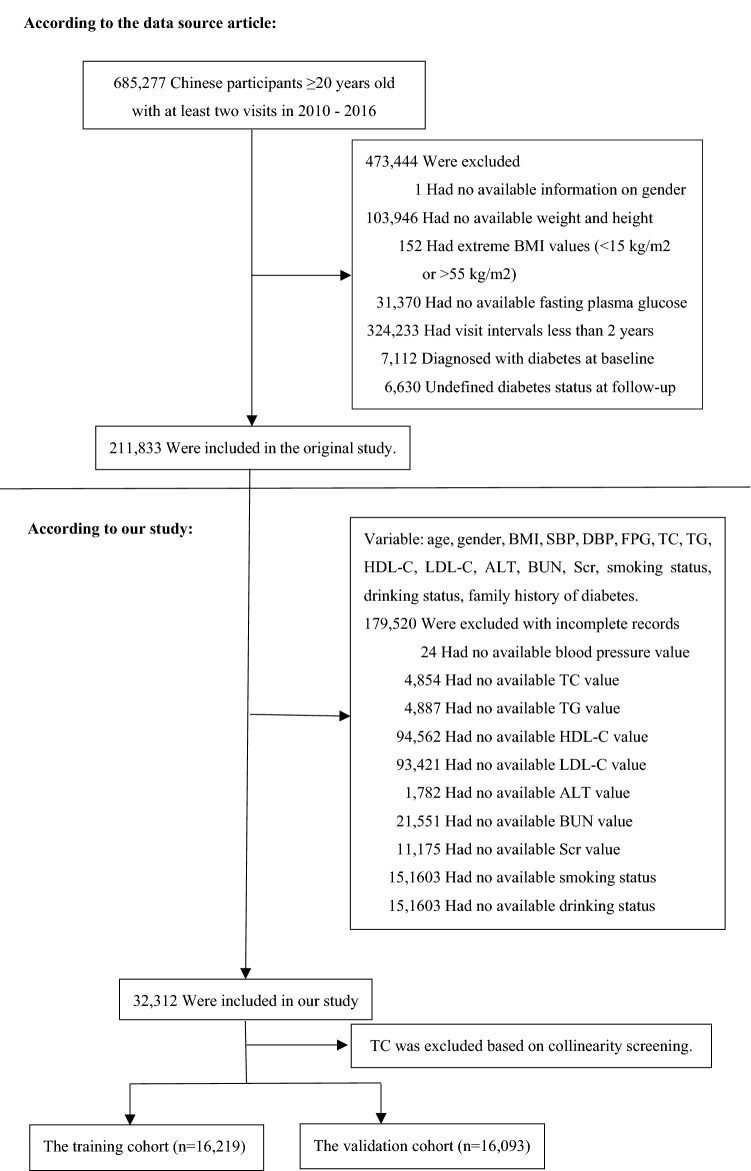
Flowchart of study participants.

The original study initially included all study participants at least 20 years old with at least two routine health checks between 2010 and 2016. Participants were excluded at baseline in the original study, as follows:(1) no available information on weight, height and gender; (2) extreme BMI values (< 15 kg/m^2^ or > 55 kg/m^2^); (3) visit intervals < 2 years; (4) no available fasting plasma glucose value; (5) participants diagnosed with diabetes at baseline (participants diagnosed by self-report or diagnosed by a fasting plasma glucose ≥ 7.0 mmol/L) and participants with undefined diabetes status at follow-up. A total of 211,833 participants remained after applying the exclusion criteria in the original study. Our study further excluded participants with the missing value of baseline variables to predict the 3-year risk of incident diabetes. Figure 1 depicted the participants' selection process. Finally, our study included 32,312 subjects (20,995 male and 11,317 female) for secondary analysis.

The study was conducted in accordance with the Declaration of Helsinki and patient consent was not required, referencing the original study article^30^.

### Variable measurement

Participants were required to do a personal questionnaire on demographic, lifestyle, medical history, and family history of chronic disease in each visit to the health check center. And trained staff conducted the baseline examination, including anthropometric measurements and laboratory biochemical measurements. Weight was measured in light clothing without shoes to the nearest 0.1 kg. The height was accurate to 0.1 cm. BMI was equal to the weight divided by the square of height, which is accurate to 0.1 kg/m^2^. And the staff used a standard mercury sphygmomanometer to measure their blood pressure. Fasting venous blood samples were taken after fasting for at least 10 h each visit. Plasma glucose levels were measured by the glucose oxidase method. The clinical measurements of FPG, TC, TG, LDL-C, HDL-C, BUN, Scr, and ALT were performed on an autoanalyzer (Beckman 5800). The data were collected under standardized conditions and conducted following uniform procedures. Laboratory methods also were carefully standardized through stringent internal and external quality controls.

### Definitions

The diabetes definitions were fasting blood glucose ≥ 7.00 mmol/L and/or self-reported diabetes during follow-up. Patients were censored either at the time of the diagnosis or at the last visit, whichever comes first.

### Statistical analysis

All participants were randomly stratified into the training cohort and the validation cohort. Baseline characteristics were expressed as means ± standard deviations (normal distribution) or medians (quartiles) (skewed distribution) for continuous variables and as frequency or percentages for categorical variables. Two-sample t-tests were applied to analyze differences between training cohort and validation cohort for normally distributed continuous variables, Wilcoxon rank-sum tests for non-normally distributed continuous variables, and chi-square tests for categorical variables. Standardized differences of less than 0.10 for a given covariate indicate a relatively small imbalance^31^. We also showed the baseline characteristics of the training and validation cohort stratified by the incidence of diabetes. After collinearity screening, logistic regression models were used to assess each variable's significance to investigate the independent risk factors of developing diabetes. The risk factors reported in the literature associated with incident diabetes were candidates for the multivariate analysis^26–28,32–35^.

To find a simple and reliable risk prediction model, we established four models for comparison. First, we apply all risk factors to build a full model. Second, we conducted a backward step-down selection process according to the Akaike information criterion (AIC) to establish a parsimonious model (stepwise model)^36^. Third, according to the multivariable fractional polynomials (MFP) algorithm, we used the iterative fashion to determine the significant variables and functional form by backward elimination to establish a stable model (MFP model) in the real world^37^. The least absolute shrinkage and selection operator (LASSO) method is suitable for reducing high-dimensional data and is applied to select the most useful prediction candidates^24,25^. Candidates with non-zero coefficients are selected to establish LASSO model^38^. Considering that fewer variables in the LASSO model and the prediction performance are relatively good, we choose the LASSO model for further analysis. To evaluate and compare the discriminatory power of these prediction models, we plotted the receiver operating characteristic (ROC) curve and calculated the area under the ROC curve (AUC) with 95% confidence intervals (CI) in the training cohort and validation cohort, respectively. We simultaneously presented the sensitivity, specificity, accuracy, positive predictive value (PPV), negative predictive value (NPV), positive likelihood ratio (PLR), negative likelihood ratio (NLR), and diagnostic odds ratio (DOR) of these four models calculated according to standard definitions. Sensitivity = True positive rate (TPR) = (Σ True positive)/(Σ Condition positive), Specificity = True negative rate (TNR) = (Σ True negative)/(Σ Condition negative), Accuracy = [ (Σ True positive) + (Σ True negative)] / (Σ Total population), Positive predictive value (PPV) = (Σ True positive)/(Σ Predicted condition positive), Negative predictive value (NPV) = (Σ True negative)/(Σ Predicted condition negative), False negative rate (FNR) = (Σ False negative)/(Σ Condition positive), False positive rate (FPR) = (Σ False positive)/(Σ Condition negative), Positive likelihood ratio (PLR) = TPR/FPR, Negative likelihood ratio (NLR) = FNR/TNR, DOR = PLR/NLR. Besides, we obtained a diabetic prediction formula for the LASSO model. The nomogram is based on proportionally converting each regression coefficient in multivariate logistic regression to a 0- to 100-point scale^39^. The effect of the variable with the highest β coefficient (absolute value) is assigned 100 points. The points are added across independent variables to derive total points, converted to predicted probabilities of developing diabetes. The nomogram score is a numeric value representing the prediction model score of the individual patient. Sensitivity and specificity for predicting diabetes at different cut-off values of nomogram scores are different. Besides, we compared the predicted risk and observed a 3-year incidence of deciles of predicted diabetes risk for the training cohort in the nomogram. The predicted and actual risks in each decile were compared by the Hosmer–Lemeshow × 2 test^40^. Decision curve analysis was conducted to determine the clinical use of the risk prediction model for diabetes: the proportion of the person who showed a true positive result subtracted by the proportion of the person who showed the false positive result, and then weighed the relative hazard of the false positive and false negative results to obtain a net benefit of making a decision^41^. Bootstraps with 500 resample were applied to ROC curve, nomogram and decision curve analysis to decrease the overfit bias^27,42^. We also performed the ROC curve to analyze each risk factor of incident diabetes' performances and optimal cut-off value in the LASSO model. What’s more, we used a cohort of 12,545 Japanese participants from the NAGALA (NAfld in the Gifu Area, Longitudinal Analysis) database for the external validation. The data were also extracted from the ‘DATADRYAD’ database (www.Datadryad.org), shared by Okamura et al.^43^ from: Ectopic fat obesity presents the greatest risk for incident type 2 diabetes: a population-based longitudinal study. Dryad Digital Repository. https://doi.org/10.1038/s41366-018-0076-3. And we did a sensitivity analysis on the overall population of the original study (n = 211,833). Multiple imputations were used to replace the missing values. All results are reported according to the TRIPOD statement^44^.

All analyses were performed with the statistical software package R (http://www.R-project.org The R Foundation) and Empower-Stats (http://www.empowerstats.com, X&Y Solutions, Inc, Boston, MA). The tests were 2‐tailed, and P < 0.05 was taken as statistically significant.

### Ethical approval

In the previously published article^29^ Ying Chen, et al. has stated the study was conducted in accordance with the Declaration of Helsinki, and the Rich Healthcare Group Review Board approved the original research, and the information was retrieved retrospectively.

## Results

The present study included 32,312 eligible participants (64.98% men and 35.02% women). Figure 1 depicted the participant's selection process. The mean age of all participants was 43.12 ± 12.62 years old. During the 2.66 years of the median follow-up period, a total of 296 participants developed diabetes. The mean BMI was 23.55 ± 3.30 kg/m^2^. The mean SBP and DBP were 119.80 ± 15.83 and 74.95 ± 10.50 mmHg, respectively. The mean FPG was 4.97 ± 0.62 mmol/L. The mean HDL-C and LDL-C were 1.34 ± 0.31 and 2.74 ± 0.69 mmol/L, respectively. We excluded TC based on collinearity screening. The mean BUN and Scr were 4.71 ± 1.17 mmol/L and 72.24 ± 15.23 umol/L, respectively. The mean follow-up period was 2.66 ± 0.42 years.

### Baseline characteristics of participants

Table 1 illustrated the basic demographic, anthropological, and clinical information of the eligible participants. We divided all participants into the training cohort (n = 16,219) and the validation cohort (n = 16,093). During the 2.66 years of the median follow-up period, 155 and 141 participants developed diabetes in the training and validation cohort, respectively. As for all baseline characteristics, the difference between the training cohort and the validation cohort was not statistically significant (all *P* > 0.05).

**Table 1:** Baseline characteristics of the training and validation cohorts.

Characteristic	Training cohort	Validation cohort	Standardized difference	*P* value
Participants	16,219	16,093		
Age (year)	43.15 ± 12.65	43.10 ± 12.59	0.00 (− 0.02, 0.03)	0.747
Gender			0.00 (− 0.02, 0.02)	0.790
Male	10,527 (64.91%)	10,468 (65.05%)		
Female	5692 (35.09%)	5625 (34.95%)		
BMI (kg/m^2^)	23.56 ± 3.28	23.54 ± 3.32	0.01 (− 0.01, 0.03)	0.527
SBP (mmHg)	119.74 ± 15.73	119.85 ± 15.94	0.01 (− 0.01, 0.03)	0.526
DBP (mmHg)	74.97 ± 10.53	74.94 ± 10.48	0.00 (− 0.02, 0.03)	0.758
FPG (mmol/L)	4.97 ± 0.62	4.97 ± 0.62	0.01 (− 0.01, 0.03)	0.528
TG (mmol/L)	1.17 (0.80–1.75)	1.17 (0.80–1.75)	0.00 (− 0.02, 0.02)	0.860
HDL-C (mmol/L)	1.34 ± 0.31	1.34 ± 0.30	0.01 (− 0.01, 0.03)	0.329
LDL-C (mmol/L)	2.74 ± 0.68	2.74 ± 0.69	0.00 (− 0.02, 0.02)	0.804
ALT (U/L)	19.60 (13.80–29.60)	19.60 (13.80–29.30)	0.01 (− 0.02, 0.03)	0.837
BUN (mmol/L)	4.71 ± 1.17	4.70 ± 1.16	0.01 (− 0.01, 0.03)	0.264
Scr (umol/L)	72.17 ± 15.24	72.30 ± 15.22	0.01 (− 0.01, 0.03)	0.457
Smoking status			0.00 (− 0.02, 0.02)	0.804
Never	12,240 (75.47%)	12,164 (75.59%)		
Ever/Current	3979 (24.53%)	3929 (24.41%)		
Drinking status			0.01 (− 0.02, 0.03)	0.621
Never	13,018 (80.26%)	12,952 (80.48%)		
Ever/Current	3201 (19.74%)	3141 (19.52%)		
Family history			0.00 (− 0.02, 0.03)	0.700
No	15,302 (94.35%)	15,199 (94.44%)		
Yes	917 (5.65%)	894 (5.56%)		

Values are n (%) or mean ± SD.

BMI, Body mass index; SBP, Systolic blood pressure; DBP, Diastolic blood pressure; FPG; Fasting plasma glucose; TG, Triglyceride; HDL-C, High density lipoprotein cholesterol; LDL-C, Low density lipid cholesterol; ALT, Alanine aminotransferase; BUN, Blood urea nitrogen; Scr, Serum creatinine; Family history, Family history of diabetes.

Table 2 showed the baseline characteristics of the two cohorts by incident diabetes status. The participants with incident diabetes had higher age, BMI, SBP, DBP, FPG, TG, ALT, BUN, Scr, and higher rates of ever or current smokers in the training and validation cohort (all *P* < 0.05). And there was no statistically significant difference in the family history of diabetes (*P* > 0.05).

**Table 2:** Baseline characteristics for the training and validation cohorts by incident diabetes status.

Characteristic	Training cohort			Validation cohort		
	No diabetes	Incident diabetes	P value	No diabetes	Incident diabetes	P value
Participants	16,064	155		15,952	141	
Age (year)	43.03 ± 12.60	55.34 ± 12.68	< 0.001	42.98 ± 12.52	56.57 ± 12.88	< 0.001
Gender			< 0.001			< 0.001
Male	10,399 (64.73%)	128 (82.58%)		10,355 (64.91%)	113 (80.14%)	
Female	5665 (35.27%)	27 (17.42%)		5597 (35.09%)	28 (19.86%)	
BMI (kg/m^2^)	23.54 ± 3.27	26.27 ± 3.17	< 0.001	23.51 ± 3.31	26.30 ± 3.39	< 0.001
SBP (mmHg)	119.61 ± 15.67	132.81 ± 16.30	< 0.001	119.76 ± 15.88	129.99 ± 19.58	< 0.001
DBP (mmHg)	74.91 ± 10.50	81.14 ± 11.17	< 0.001	74.90 ± 10.47	78.69 ± 10.67	< 0.001
FPG (mmol/L)	4.96 ± 0.61	6.03 ± 0.69	< 0.001	4.96 ± 0.61	6.01 ± 0.70	< 0.001
TG (mmol/L)	1.16 (0.80–1.74)	1.83 (1.24–2.67)	< 0.001	1.16 (0.80–1.74)	1.69 (1.11–2.60)	< 0.001
HDL-C(mmol/L)	1.34 ± 0.30	1.35 ± 0.79	0.709	1.34 ± 0.30	1.29 ± 0.30	0.071
LDL-C(mmol/L)	2.74 ± 0.68	2.92 ± 0.65	< 0.001	2.74 ± 0.69	2.81 ± 0.71	0.202
ALT(U/L)	19.50 (13.70–29.40)	26.70 (19.00–43.90)	< 0.001	19.50 (13.80–29.10)	27.10 (18.90–40.60)	< 0.001
BUN (mmol/L)	4.71 ± 1.17	5.15 ± 1.43	< 0.001	4.69 ± 1.16	5.16 ± 1.33	< 0.001
Scr (umol/L)	72.15 ± 15.21	74.77 ± 17.83	0.033	72.28 ± 15.20	74.81 ± 16.80	0.049
Smoking status			< 0.001			< 0.001
Never	12,150 (75.63%)	90 (58.06%)		12,084 (75.75%)	80 (56.74%)	
Ever/Current	3914 (24.37%)	65 (41.94%)		3868 (24.25%)	61 (43.26%)	
Drinking status			0.012			0.335
Never	12,906 (80.34%)	112 (72.26%)		12,834 (80.45%)	118 (83.69%)	
Ever/Current	3158 (19.66%)	43 (27.74%)		3118 (19.55%)	23 (16.31%)	
Family history			0.139			0.124
No	15,160 (94.37%)	142 (91.61%)		15,070 (94.47%)	129 (91.49%)	
Yes	904 (5.63%)	13 (8.39%)		882 (5.53%)	12 (8.51%)	

Values are n (%) or mean ± SD.

SD, Standardized difference; BMI, Body mass index; SBP, Systolic blood pressure; DBP, Diastolic blood pressure; FPG; Fasting plasma glucose; TG, Triglyceride; HDL-C, High-density lipoprotein cholesterol; LDL-C, Low-density lipid cholesterol; ALT, Alanine aminotransferase; BUN, Blood urea nitrogen; Scr, Serum creatinine; Family history, Family history of diabetes.

### Univariate and multivariate analysis

Table 3 displayed risk predictors for incident diabetes in the univariate and multivariate logistic regression analysis. The univariate analysis showed that age (OR = 1.066), female (OR = 0.421), BMI (OR = 1.238), SBP (OR = 1.039), DBP (OR = 1.042), FPG (OR = 13.925), TG (OR = 1.304), LDL-C (OR = 1.303), ALT (OR = 1.010), BUN (OR = 1.343), Scr (OR = 1.011), ever/current smoking (OR = 2.308) and family history of diabetes (OR = 1.561) was associated with incident diabetes (all *P* < 0.05), HDL-C, and drinking status were not correlated with diabetes (all *P* > 0.05). The multivariate analysis showed that age (OR = 1.047), BMI (OR = 1.122), FPG (OR = 8.564), HDL-C (OR = 1.515), ALT (OR = 1.008), ever/current smoking (OR = 1.527), and family history of diabetes (OR = 1.902) were associated with incident diabetes (all *P* < 0.05). However, gender, SBP, DBP, TG, LDL-C, BUN, Scr, and drinking status was not correlated with diabetes (all *P* > 0.05).

**Table 3:** Risk predictors **for incident diabetes in the univariate and multivariate analysis.**

Variable	Univariate (OR,95%CI, P)	Multivariate (OR,95%CI, P)
Age(year)	1.066 (1.058, 1.075) < 0.00001	1.047 (1.036, 1.058) < 0.00001
**Gender**		
Male	1.0	1.0
Female	0.421 (0.314, 0.564) < 0.00001	0.675 (0.451, 1.009) 0.05506
BMI (kg/m^2^)	1.238 (1.202, 1.274) < 0.00001	1.122 (1.077, 1.168) < 0.00001
SBP (mmHg)	1.039 (1.033, 1.046) < 0.00001	1.008 (0.999, 1.018) 0.07860
DBP (mmHg)	1.042 (1.032, 1.052) < 0.00001	0.994 (0.980, 1.009) 0.42703
FPG (mmol/L)	13.925 (11.487, 16.882) < 0.00001	8.564 (6.978, 10.509) < 0.00001
TG (mmol/L)	1.304 (1.238, 1.373) < 0.00001	1.069 (0.994, 1.150) 0.07091
HDL-C (mmol/L)	0.831 (0.567, 1.216) 0.34028	1.515 (1.101, 2.086) 0.01085
LDL-C (mmol/L)	1.303 (1.115, 1.524) 0.00090	0.858 (0.722, 1.020) 0.08233
ALT (U/L)	1.010 (1.007, 1.012) < 0.00001	1.008 (1.004, 1.011) 0.00016
BUN (mmol/L)	1.343 (1.232, 1.464) < 0.00001	1.026 (0.924, 1.139) 0.63007
Scr (umol/L)	1.011 (1.004, 1.018) 0.00368	0.992 (0.982, 1.002) 0.10641
**Smoking status**		
Never	1.0	1.0
Ever/Current	2.308 (1.831, 2.910) < 0.00001	1.527 (1.158, 2.014) 0.00271
**Drinking status**		
Never	1.0	1.0
Ever/Current	1.177 (0.894, 1.550) 0.24580	0.822 (0.606, 1.115) 0.20821
**Family history**		
No	1.0	1.0
Yes	1.561 (1.034, 2.359) 0.03421	1.902 (1.219, 2.967) 0.00461

BMI, Body mass index; SBP, Systolic blood pressure; DBP, Diastolic blood pressure; FPG; Fasting plasma glucose; TG, Triglyceride; HDL-C, High-density lipoprotein cholesterol; LDL-C, Low-density lipid cholesterol; ALT, Alanine aminotransferase; BUN, Blood urea nitrogen; Scr, Serum creatinine; Family history, Family history of diabetes.

OR, Hazard ratios; CI, Confidence interval; Ref, Reference.

### Development and validation of risk prediction models

**Figure 2:**
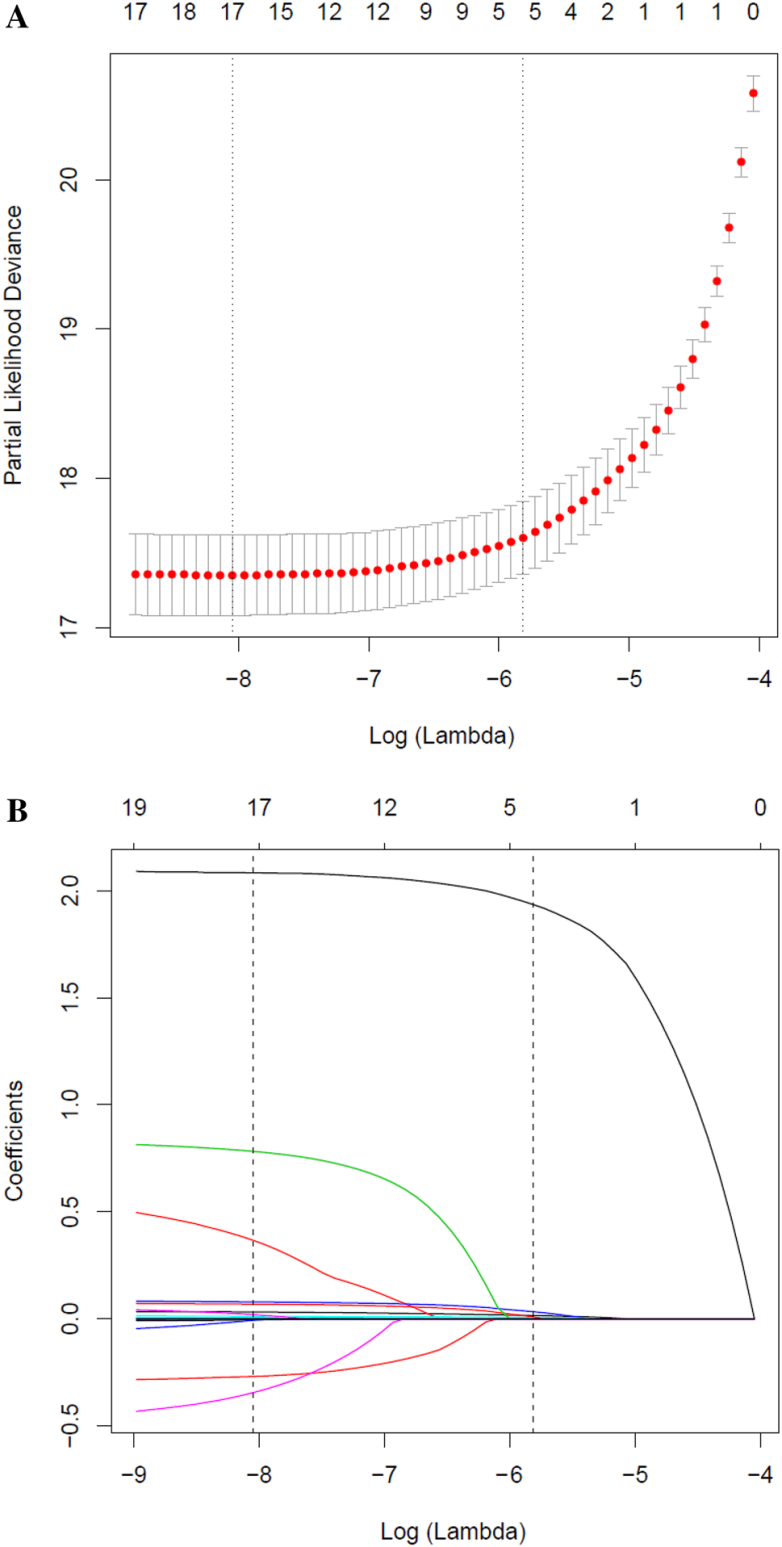
Risk predictors selection using the LASSO logistic regression model. (**A**) Optimal predictor (lambda) selection in the LASSO model with fivefold cross validation by minimum criteria. The area under the receiver operation characteristic curve was plotted versus log (lambda). Dotted vertical lines were drawn at the optimal values by using the minimum criteria and the 1 SE of the minimum criteria; (**B**) LASSO coefficient profiles of the 15 predictors. A coefficient profile plot was developed against the log (lambda) sequence. Vertical line was drawn at the value selected with fivefold cross validation, where optimal lambda resulted in 5 predictors with nonzero coefficients (lambda = 0.003).

**Figure 3:**
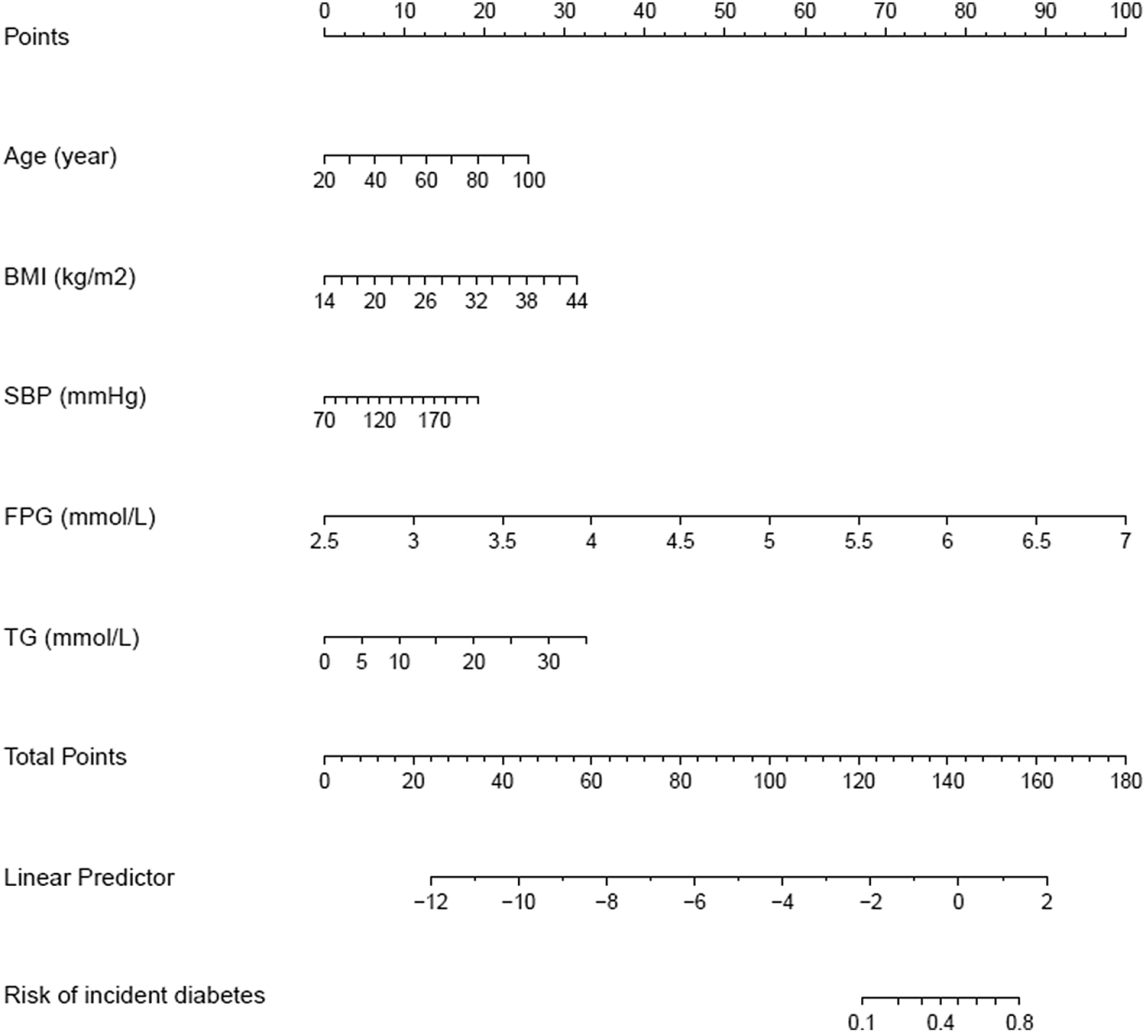
Nomogram to predict the risk of diabetes for Chinese adults. The patient’s score for each risk predictor is plotted on the appropriate scale. The patient’s score for each risk predictor is plotted on the appropriate scale and vertical lines are drawn from that value to the top Points scale to obtain the corresponding scores. All scores are summed to obtain the total points score. The total points score is plotted on the bottom Total Points scale. The corresponding value shows the predicted probability of incident diabetes.

We established four prediction models, including the full model, stepwise model, MFP model and LASSO model. 15 risk factors were reduced to 5 potential risk predictors based on the training cohort (Fig. 2A,B) that had nonzero coefficients in the LASSO model, which were less than the other three models. These potential risk predictors were age, BMI, SBP, FPG and TG. In the training cohort, AUCs of the LASSO model, full model, stepwise model and MFP model were 0.9125, 0.9155, 0.9161 and 0.9161. In the validation cohort, AUCs of the LASSO model, full model, stepwise model and MFP model were 0.9030, 0.9146, 0.9131 and 0.9131, respectively (Table 4, Table S1). The AUC of these four models were relatively close. Given that the LASSO model incorporated fewer risk factors and could predict the 3-year diabetes risk relatively well, we choose the LASSO model as the final risk prediction model for diabetes and further construct a corresponding nomogram (Fig. 3). The total nomogram score was applied to obtain the sort of probability for predicting incident diabetes. The 3-year diabetes probability was calculated by: − 23.14183 + 0.03224* age (year) + 0.10645* BMI (kg/m^2^) + 0.01388* SBP (mmHg) + 2.24841* FPG (mmol/L) + 0.09444* TG (mmol/L).

**Table 4:** Prediction performance of the nomogram for the risk of diabetes.

	AUC	95% CI		Best threshold	Specificity (%)	Sensitivity (%)	Accuracy (%)	PPV (%)	NPV (%)	PLR	NLR	DOR
		Lower	Upper									
Training cohort	0.9125	0.8887	0.9364	0.0072	80.11	89.03	80.20	4.14	99.87	4.4764	0.1369	32.6967
Validation cohort	0.9030	0.8747	0.9313	− 4.8295	82.30	85.11	82.33	4.08	99.84	4.8091	0.1810	26.5756

AUC, Area under curve; CI, Confidence interval; PPV, Positive predictive value; NPV, Negative predictive value; PLR, Positive likelihood ratio; NLR, Negative likelihood ratio; DOR, Diagnostic odds ratio.

The algorithm of diabetes risk in LASSO model:

Model = − 23.14183 + 0.03224* age (year) + 0.10645* BMI (kg/m^2^) + 0.01388* SBP (mmHg) + 2.24841* FPG (mmol/L) + 0.09444* TG (mmol/L).

### Prediction performance of the LASSO model

**Figure 4:**
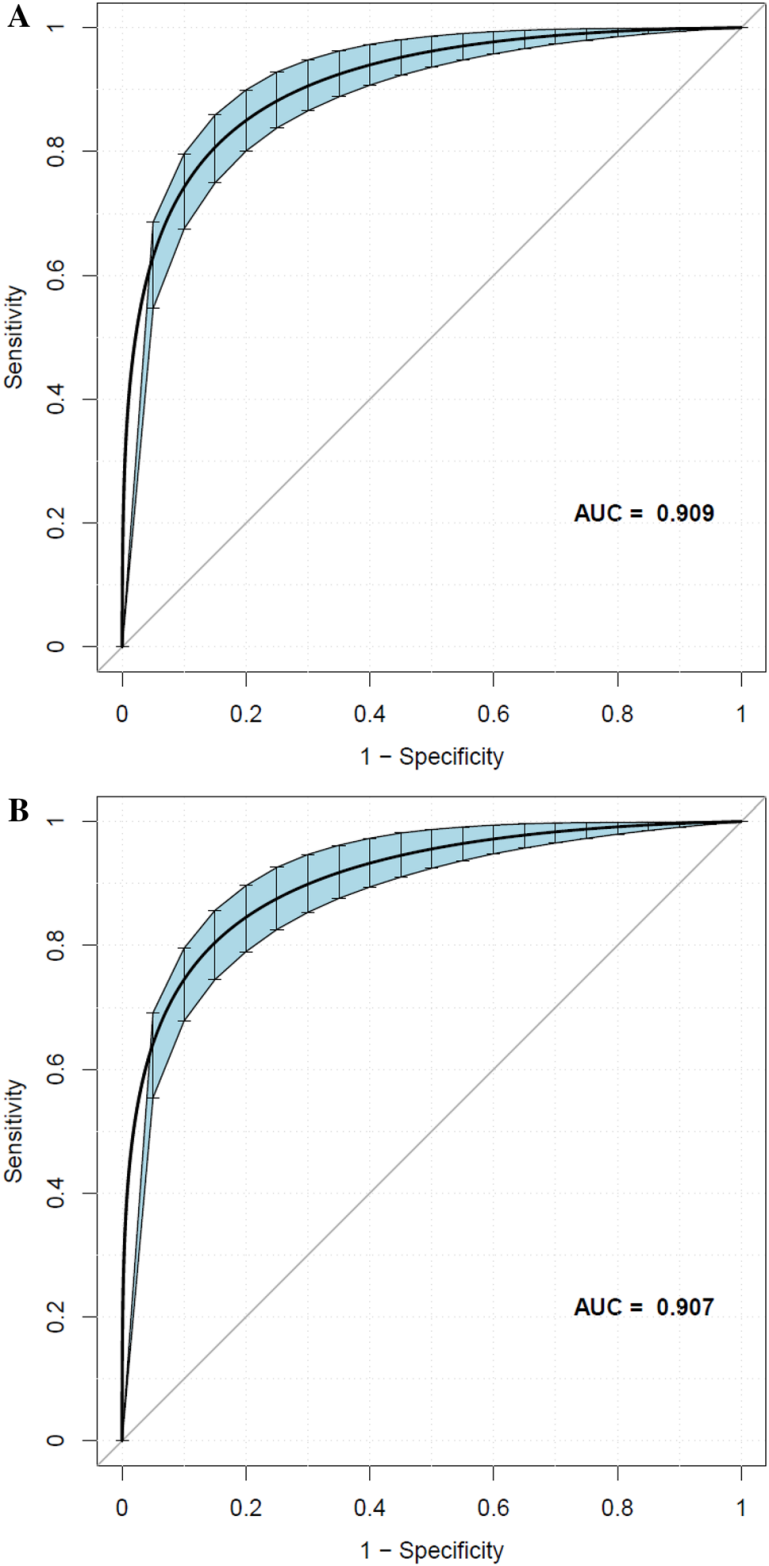
Using bootstrap resampling validation (times = 500) to confirm the prediction performance stability of the nomogram in the training cohort (**A**) and validation cohort (**B**).

In the training cohort and the validation cohort, AUCs of the LASSO model were 0.9125 (95% CI, 0.8887–0.9364) and 0.9030 (95% CI, 0.8747–0.9313), respectively (Table 4). At the best threshold, the sensitivity rates were 89.03% and 85.11%, and the specificity percentages were 80.11% and 82.30% for the training cohort and the validation cohort, respectively. Notably, the AUC of the prediction nomogram was internally confirmed to be relatively stable through the bootstrap validation (AUC = 0.909) (Fig. 4). The differences in AUC, sensitivity, specificity, and accuracy between the four models were relatively small, both in the training cohort and the validation cohort. The other three models' results were shown in the Supplemental Appendix (Table 4, Table S1, Fig S1).

**Figure 5:**
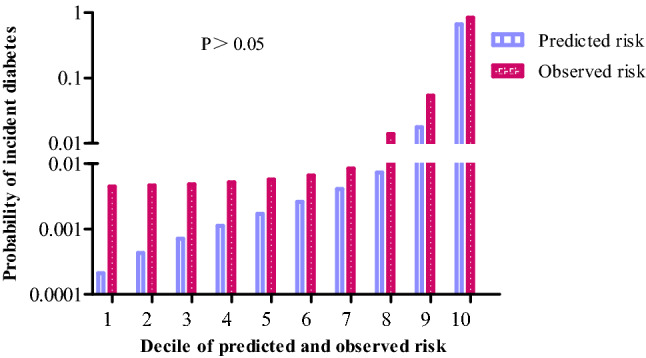
Comparison between predicted and observed 3-year incidence of deciles of predicted diabetes risk score for the training cohort in the nomogram.

We also evaluated how close the predicted risk was to the observed 3-year incidence of deciles of predicted diabetes risk for the nomogram's training cohort. Figure 5 illustrates the fraction of individuals in each decile of predicted risk in the training cohort. Our nomogram underestimated the 3-year risk of diabetes. However, the Hosmer–Lemeshow × 2 test showed no statistically significant difference between the predicted diabetes risk and observed diabetes (*P* > 0.05).

We also showed the prediction performance of each risk predictor in the nomogram, including age, BMI, SBP, FPG, TG (Table S2, Fig S2). The AUC of the prediction nomogram was greater than the AUC of each risk factor for incident diabetes. The predictive ability of other similar risk prediction models for diabetes in China was summarized in Table S3.

### Optimal cut-off value for nomogram score

Table 5 showed the sensitivity and specificity for predicting diabetes at different cut-off values. At a cut-off value of 0.05, the specificity is 95.61% and the sensitivity is 61.29%. When the cut-off value increased to 0.3, the specificity increased to 99.78%, while the sensitivity drops to 12.26%. In summary, although higher cut-off values resulted in higher specificity, the sensitivity rapidly fell to a relatively low point.

**Table 5:** Values of sensitivity, specificity and predictive values of the nomogram scores at different cut-off values.

Predicted probability	Specificity (%)	Sensitivity (%)	Accuracy (%)	PPV (%)	NPV (%)	PLR	NLR	DOR
≥ 0.05	95.61	61.29	95.28	11.86	99.61	13.95	0.40	34.44
≥ 0.10	97.43	43.87	96.92	14.14	99.45	17.06	0.58	29.62
≥ 0.15	98.62	32.90	98.00	18.75	99.35	23.92	0.68	35.15
≥ 0.20	99.10	24.52	98.39	20.88	99.27	27.35	0.76	35.91
≥ 0.25	99.60	16.77	98.80	28.57	99.20	41.46	0.84	49.61
≥ 0.30	99.78	12.26	98.95	35.19	99.16	56.26	0.88	63.98
≥ 0.35	99.88	7.74	99.00	37.50	99.12	62.18	0.92	67.32
≥ 0.40	99.94	3.87	99.02	37.50	99.08	62.18	0.96	64.65
≥ 0.45	99.98	1.29	99..04	40.00	99.06	69.09	0.99	69.98
≥ 0.50	99.99	1.29	99.04	50.00	99.06	103.64	0.99	104.98

PPV, Positive predictive value; NPV, Negative predictive value; PLR, Positive likelihood ratio; NLR, Negative likelihood ratio; DOR, Diagnostic odds ratio.

### Clinical use of the nomogram

**Figure 6:**
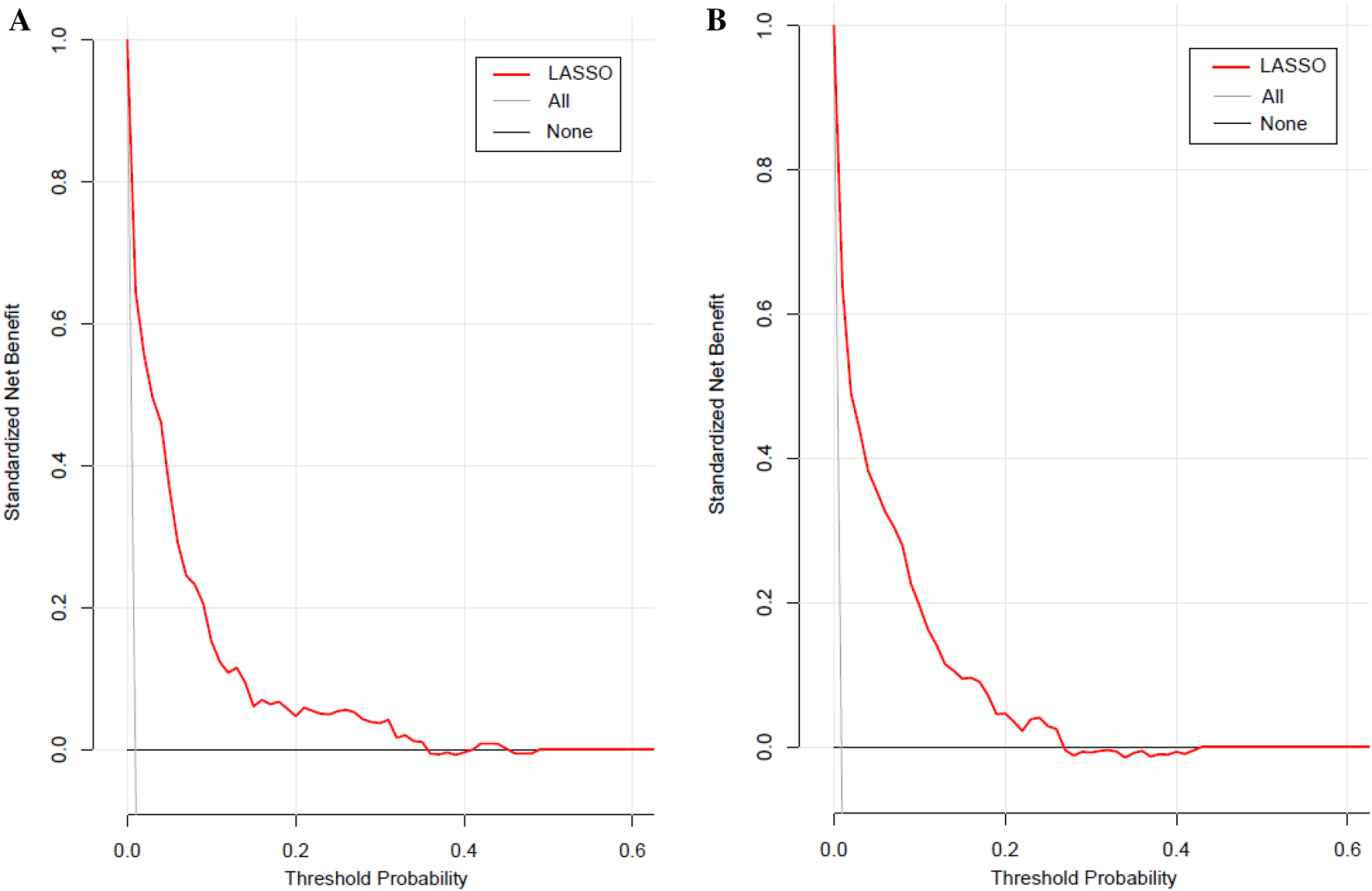
The decision curve analysis of the LASSO model for 3-year diabetes risk in the training cohort (**A**) and validation cohort (**B**). The black line represents the net benefit when none of the participants are considered to develop diabetes, while the light gray line represents the net benefit when all participants are considered to develop diabetes. The area between the "no treatment line" (black line) and "all treatment line" (light gray line) in the model curve indicates the clinical utility of the model. The farther the model curve is from the black and light gray lines, the better the clinical use of the nomogram. (Using bootstraps with 500 resamples).

Figure 6 demonstrated the result of the LASSO model's decision curve analysis in the training and validation cohorts. The black line represents the net benefit when none of the participants are considered to develop diabetes. In contrast, the light gray line represents the net benefit when all participants are considered to develop diabetes. The area between the "no treatment line" (black line) and "all treatment line" (light gray line) in the model curve indicates the clinical utility of the model. The farther the model curve is from the black and light gray lines, the better the nomogram's clinical application. Specifically, in the training cohort, if the threshold probability of a patient was 4% in the LASSO model, the net benefit was about 50%, which was equivalent to performing 50 additional diabetes screenings (such as oral glucose tolerance test) per 100 Chinese adults when without a significant change in the incidence of diabetes.

### External validation

**Figure 7:**
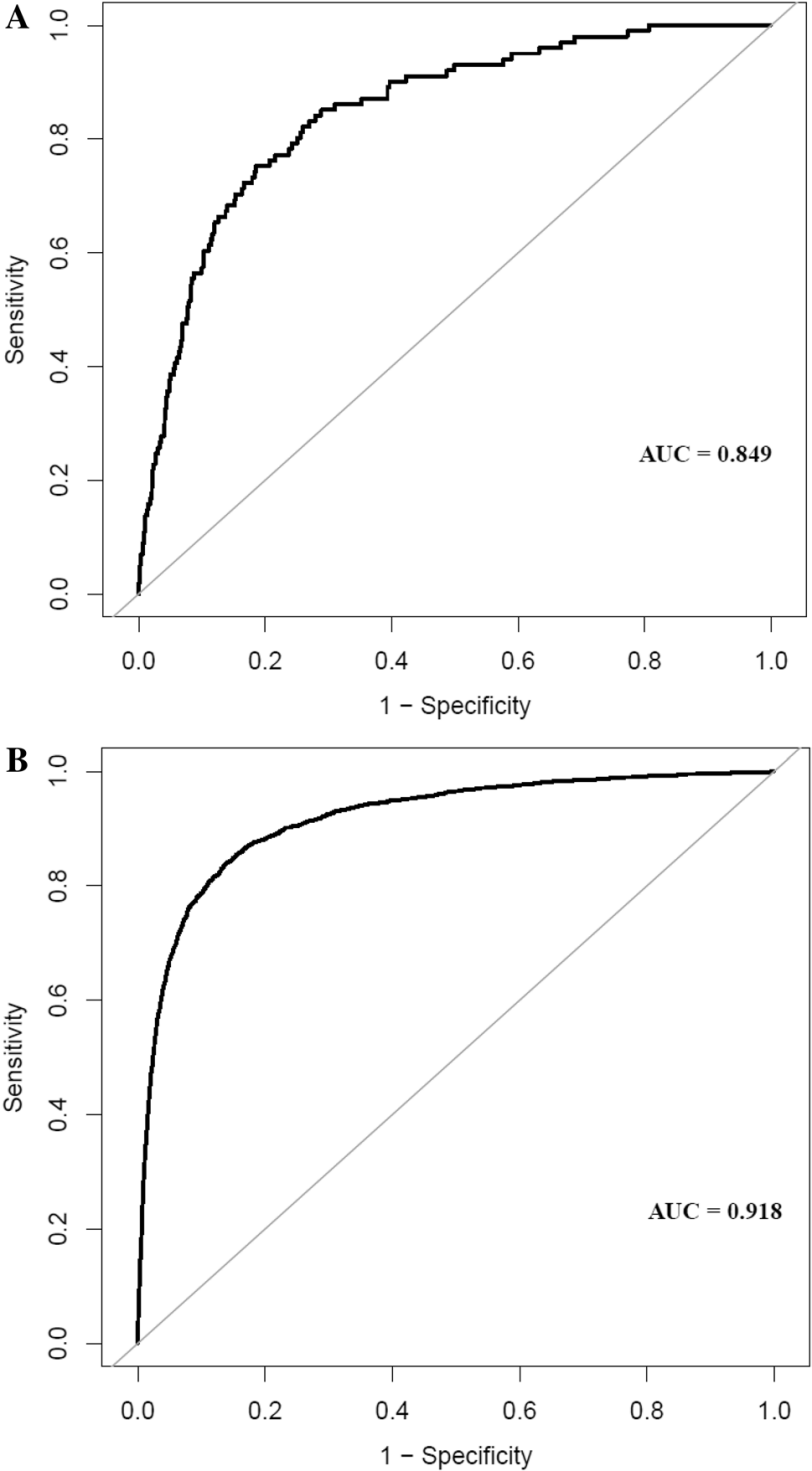
The ROC curves of the nomogram in the external validation cohort (**A**) the overall population of the original study (**B**).

The external validation was performed on a cohort of 12,545 Japanese participants. The mean age, BMI, SBP, and FPG of the participants were 43.56 ± 8.68 years old, 22.11 ± 3.11 kg/m^2^, 114.42 ± 14.89 mmHg, and 5.15 ± 0.41 mmol/L, respectively. The median TG was 0.75 (0.50–1.12) mmol/L. (Table S4).The AUC of the external validation was 0.849 (Fig. 7A). At the best threshold, the specificity and sensitivity rates were 81.46% and 75.25%, respectively. (Table S5). The external validation revealed that our nomogram had excellent prediction performance.

### Sensitivity analysis

To perform the LASSO model's sensitivity analysis, we used multiple imputations to replace the missing values of variables of the overall population in the original study (n = 211,833). The mean age, BMI, SBP, and FPG were 42.10 ± 12.65 years old, 23.24 ± 3.34 kg/m^2^, 119.06 ± 16.38 mmHg, and 4.92 ± 0.61 mmol/L, respectively. The median of TG was 1.07 (0.73–1.62). (Table S4). The AUC was 0.918 (Fig. 7b). At the best threshold, the specificity and sensitivity rates were 86.17% and 83.90%, respectively. (Table S5).

## Discussion

In this retrospective cohort study, we developed and validated a personalized prediction nomogram for the 3-year risk of incident diabetes by cost-effective and readily available parameters among Chinese adults, helping clinicians identify individuals with a high risk of developing diabetes. The nomogram included five parameters: age, BMI, SBP, FPG, and TG. The internal and external validation showed that our nomogram had excellent prediction performance. We also summarized the sensitivity and specificity of the nomogram for predicting diabetes at different cut-off values. Decision curve analysis illustrated the clinical use of the nomogram.

Although many diabetes risk prediction models based on demographic, anthropological, and clinical information have been established, they are mainly used in European^45–47^and American populations^48–50^. Only a limited number of reliable diabetes prediction models were established in the Chinese population, each of which included different risk predictors. Besides, their prediction performance and clinical usefulness varied greatly. In 2019, Zeyin Lin et al.^51^ performed cox proportional hazards regression analysis to develop a nomogram to predict the 5-year incidence of type 2 diabetes mellitus based on age, sex, BMI, and hypertension dyslipidemia, smoking status and family history of diabetes. The C-index of the model was 0.815 (95% CI, 0.797–0.834). However, they did not conduct a decision curve analysis to evaluate the clinical usefulness of the model. Additionally, they did not try other methods to compare and screen the most suitable risk prediction model for incident diabetes. Moreover, age, BMI, TC, TG, HDL-C, and LDL-C are continuous risk predictors, and categorizing them into categories will cause detrimental information loss and affect the ability to detect real relationships^52,53^. In 2019, Kun Wang et al.^54^ developed a nomogram to predict the 3-year risk of T2DM in healthy mainland China residents based on age, BMI, FPG, LDL-C, HDL-C, and TG. The AUCs were 0.847 (95% CI, 0.801–0.892) and 0.755(95% CI, 0.717–0.794) for females and males, respectively. Consistent with our nomogram, their nomogram incorporated continuous predictors. Besides, they established a full model, MFP model, and stepwise model, and chose an appropriate model after comparison. However, they did not take into account family history of diabetes, smoking, and drinking history. Although our nomogram did not include them, we have considered them in the variable selection process. Besides, they did not measure how closely the predicted risk fits the actual risk. In 2015, Carlos et al.^55^ developed a simple non-laboratory- and laboratory-based risk assessment algorithms and nomogram to predict undiagnosed diabetes in Hong Kong. The AUCs were 0.686 (95% CI, 0.650–0.722) for non-laboratory-based algorithm and 0.696 (95% CI, 0.661–0.731) for laboratory-based algorithm. They produced two different nomograms based on anthropometric and biochemical assessments, respectively. And each nomogram included relatively few risk predictors, which may lead to insufficient accuracy and prediction performance of the diabetes prediction model. Thus, their model's predictive ability is relatively low (AUC = 0.686 and 0.696), which revealed that we need to incorporate relatively more risk factors in developing the risk prediction model to ensure the prediction performance. Furthermore, this was a single-center study based on a professional driver community project. The cohort's inappropriate selection and relatively small sample size made it insufficient to represent the Chinese population. It is worth mentioning that none of these studies have performed external validation. Compared with the similar studies mentioned above, our nomogram filled those gaps. Our research sample size was considerable (n = 32,312), and participants were from multiple centers, so our findings may be better applied to the Chinese population. Unlike most previous Chinese DM risk scores with integer points or segmented values in China, our nomogram uses continuous variables to provide more precise and personalized risk prediction. It is worth mentioning that we constructed four models and selected the simplest and reliable LASSO model to ensure clinical practicality. Given that a nomogram could provide accurate and individualized risk prediction for each individual. According to the LASSO model, we constructed the corresponding nomogram, which makes up for the deficiencies of many other similar Chinese studies. Notably, our nomogram has an excellent prediction performance (AUC = 0.9125, 95% CI, 0.8887–0.9364). Besides, we proved no significant difference between the predicted diabetes risk and the observed incidence of diabetes.

Diabetes can cause various complications, bring severe physical and psychological distress to patients, and bring a huge burden to the healthcare system. And it tends to be undiagnosed due to the lack of specific symptoms. However, screening for diabetes through oral glucose tolerance test may increase the yield and economic efficiency of screening^56^. In this study, we used the LASSO model with relatively good predictive performance to construct the nomogram. And we provided a corresponding formula to calculate the risk of diabetes based on risk predictors, which could help clinicians accurately identify individuals at high risk for diabetes, guide them in timely diabetes screening, and avoid the costs and efforts of prevention and treatment in low-risk groups. And our nomogram underestimated the 3-year risk of diabetes, so the individuals at high risk of developing diabetes identified by our nomogram are indeed at higher risk. Our nomogram items are routine clinical variables readily available to clinicians, thus allowing the nomogram to be easily adopted in practice. Furthermore, the nomogram's predictive performance was high both in the internal and external validation, which suggests its high generalizability. Notably, there were subtle differences between the AUC of our model and that of internal and external validation models. AUC of the external validation model was slightly smaller than the AUC of our nomogram (AUC = 0.849 vs. AUC = 0.913). The difference may come from the following: (1) the study populations were different, our study was performed on the Chinese, and the validation dataset was from Japanese. (2) Participants with FPG ≥ 6.1 mmol/L were excluded from the external validation cohort. (3) The outcome of the external validation cohort was T2DM. However, we could not distinguish between type 1, type 2, and other diabetes types in our model. (4) Diabetes was diagnosed as HbA1c ≥ 6.5%, FPG ≥ 7 mmol/L, or self-reported in the external validation cohort. However, the definitions of diabetes in our nomogram did not include HbA1c ≥ 6.5%. For sensitivity analysis, the AUC for the original study's overall population was close to that of our nomogram (AUC = 0.918 vs. AUC = 0.913), which showed that our study participants could represent the general population.

The risk predictors included in our nomogram were age, BMI, SBP, FPG and TG, which were also included in previous diabetes risk prediction models. Venerable age is a nonmodifiable risk factor for developing diabetes^57^. Aging pancreatic β cells result in the decline of glucose sensitivity and insulin secretory defects^58^. Age-related glucose intolerance is usually accompanied by insulin resistance and β-cell dysfunction^59^. Obesity could increase the fat content of the liver and pancreas, which affect the function of pancreatic β cells^60^. Besides, obesity leads to metabolic derangements and adipose organ dysfunction, leading to insulin resistance^61^. Hypertension and diabetes are often concurrent. The substantial mediators could involve inflammation, oxidative stress, endothelial dysfunction, and insulin resistance^62^. FPG is an independent risk factor of the onset of diabetes, and people with relatively high FPG had a higher risk score of diabetes in our nomogram. It may be that FPG is closely related to insulin response and insulin sensitivity^63^. Dyslipidemia and diabetes often co-exist in the same individual. As an endocrine organ, adipose tissue can affect glucose and lipids' metabolism, and TG is the most abundant lipid in adipose tissue^64^. Excess fatty tissue can release many lipid metabolites, proinflammatory cytokines, and cellular stress, which mediate insulin resistance^65^. Therefore, the application of the five risk predictors in our models is well-founded.

There are some strengths in the present study, as follows: (1) The present study has a large sample size, and participants were from multiple centers. (2) We established four prediction models, including the LASSO model, full model, stepwise, and MFP models. And we selected the simplest LASSO model with relatively good prediction performance to construct the nomogram to ensure clinical practicability. (3) We provided a formula to calculate the risk of diabetes based on risk predictors, which helps clinicians quickly and accurately calculate the individual’s risk of developing diabetes and provide external verification information for other similar studies. (4) Our decision curve analysis demonstrated the nomogram's clinical use and could avoid performing additional diabetes screenings (such as OGTT) for individuals with low-risk diabetes. (5) We performed both internal and external validation to ensure the reliability of the results. (6) As this was a retrospective cohort study, it decreased the risk of selection bias and message bias.

Although our nomogram performed well, the study still has some potential limitations. First of all, this is a secondary retrospective study. The raw data did not provide other diabetes risk factors, such as waist/hip ratio, medical history, and lifestyle factors, affecting the onset of diabetes. However, our nomogram has excellent prediction performance in both internal and external validation, suggesting that the nomogram based on the existing five risk factors has high generalizability. Second, the database did not distinguish between type 1, type 2, and other diabetes types. And the risk factors of different kinds of diabetes are somewhat different. However, type 2 diabetes is the most common kind of diabetes, accounting for over 90% of diabetes cases^66^. The nomogram is approximately used to predict the 3-year risk of developing type 2 diabetes. Third, the researchers did not conduct an oral glucose tolerance test and measure glycosylated hemoglobin. A study showed that 55% of diabetic patients were diagnosed by testing fasting blood glucose alone in Asians^67^. Thus, the diagnostic criteria for diabetes in our study may underestimate the true prevalence of diabetes. In other words, the development and validation datasets included only very small numbers of diabetes cases, which may be related to the diagnostic criteria for diabetes in our study. However, a 2-h oral glucose tolerance test for all participants was not feasible in such a large cohort. Fourth, we excluded participants with incomplete records for complete-case analysis to build the models, which may introduce selection bias. However, we used multiple imputations to replace missing values to do sensitivity analysis. And the results proved that our study participants could well represent the overall population. Therefore, in the future, we can consider designing our studies or cooperating with other researchers to collect as many variables as possible, reduce missing values, and distinguish the types of diabetes. Fifth, there were no interactions between the covariates included within the full model, which may cause bias in the results of the full model. However, we focused predominantly on the LASSO model, which has the fewest variables and is more convenient for clinical application, rather than the full model.

## Conclusion

We developed and validated a personalized prediction nomogram for the 3-year risk of incident diabetes among Chinese adults, including age, BMI, SBP, FPG and TG. The nomogram had excellent prediction performance in both training and validation cohorts for estimating the risk of developing diabetes, and it has high generalizability. The nomogram was a simple and reliable tool to help clinicians accurately identify individuals with high diabetes risk.

## Supplementary Information

Supplementary Information.

## Data Availability

Data can be downloaded from the ‘DATADRYAD’ database (www.Datadryad.org), shared by Chen et al.^29^ from: Association of body mass index and age with incident diabetes in Chinese adults: a population-based cohort study. Dryad Digital Repository. http://dx.doi.org/10.1136/bmjopen-2018-021768.

## Abbreviations

BMI: Body mass index
SBP: Systolic blood pressure
DBP: Diastolic blood pressure
FPG: Fasting plasma glucose
TC: Total cholesterol
TG: Triglyceride
HDL-C: High-density lipoprotein cholesterol
LDL-C: Low-density lipid cholesterol
ALT: Alanine aminotransferase
BUN: Serum urea nitrogen
Scr: Serum creatinine
T2DM: Type 2 diabetes mellitus
DM: Diabetes mellitus
LASSO: Least absolute shrinkage and selection operator
SD: Standardized difference
HR: Hazard ratios
CI: Confidence intervals
Ref: Reference
PPV: Positive predictive value
NPV: Negative predictive value
PLR: Positive likelihood ratio
NLR: Negative likelihood ratio
DOR: Diagnostic odds ratio
ROC: Receiver operating characteristic
AUC: Area under the curve
