# Supplementary material for: A prediction nomogram for the 3-year risk of incident diabetes among Chinese adults
Source: Sci Rep. 2020 Dec 10;10:21716. doi: 10.1038/s41598-020-78716-1 (PMC7729957; doi:10.1038/s41598-020-78716-1)
Supplement: Supplementary file 1 — Supplementary Information. [file 41598_2020_78716_MOESM1_ESM.docx]

**A prediction nomogram for** **the 3-year risk of incident diabetes** **among Chinese adults**

**Yang Wu^1,3,5#^, Haofei Hu^2,4,5#^, Jinlin Cai^1,3,6^, Runtian Chen ^1,3,5^, Xin Zuo ^7^, Heng Cheng^7^, Dewen Yan^1,3,5*^**

^1^Department of Endocrinology, The First Affiliated Hospital of Shenzhen University, Shenzhen 518035, Guangdong Province, China

^2^Department of Nephrology, The First Affiliated Hospital of Shenzhen University, Shenzhen 518035, Guangdong Province, China

^3^Department of Endocrinology, Shenzhen Second People’s Hospital, Shenzhen 518035, Guangdong Province, China

^4^Department of Nephrology, Shenzhen Second People’s Hospital, Shenzhen 518035, Guangdong Province, China

^5^Shenzhen University Health Science Center, Shenzhen 518071, Guangdong Province, China

^6^ Shantou University Medical College, Shantou 515000, Guangdong Province, China

^7^ Department of Endocrinology, Shenzhen Third People’s Hospital, Shenzhen 518116, Guangdong Province, China

Yang Wu and Haofei Hu have contributed equally to this work.

*Corresponding author

Dewen Yan

Department of Endocrinology,

The First Affiliated Hospital of Shenzhen University,

No.3002 Sungang Road, Futian District,

Shenzhen 518035,

Guangdong Province,

China

Tel: +86-755-83366388

E-mail: ydewenyy@163.com

**Table S1 Prediction performance of** **MFP, full and stepwise model for the risk of diabetes.**

|  | Training  Full  model | cohort  Stepwise  model | MFP  model | Validation  Full  model | cohort  Stepwise  model | MFP  model |
| --- | --- | --- | --- | --- | --- | --- |
| AUC | 0.9155 | 0.9161 | 0.9161 | 0.9146 | 0.9131 | 0.9131 |
| 95% CI |  |  |  |  |  |  |
| Lower | 0.8925 | 0.8935 | 0.9011 | 0.8895 | 0.8872 | 0.8872 |
| Upper | 0.9385 | 0.9387 | 0.9419 | 0.9398 | 0.9389 | 0.9389 |
| Best threshold | 0.0122 | 0.0106 | 0.0106 | 0.0067 | 0.0075 | 0.0075 |
| Specificity, % | 87.15 | 85.70 | 85.49 | 80.80 | 82.37 | 82.37 |
| Sensitivity, % | 83.23 | 84.52 | 85.16 | 88.65 | 87.23 | 87.23 |
| Accuracy, % | 87.11 | 85.69 | 85.49 | 80.87 | 82.41 | 82.41 |
| PPV, % | 5.88 | 5.40 | 5.36 | 3.92 | 4.19 | 4.19 |
| NPV, % | 99.81 | 99.83 | 99.83 | 99.88 | 99.86 | 99.86 |
| PLR | 6.4743 | 5.9106 | 5.8689 | 4.6170 | 4.9486 | 4.9486 |
| NLR | 0.1925 | 0.1807 | 0.1736 | 0.1404 | 0.1550 | 0.1550 |
| DOR | 33.6351 | 32.7144 | 33.8119 | 32.8747 | 31.9310 | 31.9310 |

MFP, multivariable fractional polynomials; AUC, Area under curve; CI, Confidence interval; PPV, Positive predictive value; NPV, Negative predictive value; PLR, Positive likelihood ratio; NLR, Negative likelihood ratio; DOR, Diagnostic odds ratio.

**Table S2 Prediction performance of risk predictors in our nomogram.**

|  | Age | Training  BMI | cohort  SBP | FPG | TG | Age | Validation  BMI | cohort  SBP | FPG | TG |
| --- | --- | --- | --- | --- | --- | --- | --- | --- | --- | --- |
| AUC | 0.7590 | 0.7306 | 0.7254 | 0.8758 | 0.7091 | 0.7779 | 0.7303 | 0.6614 | 0.8643 | 0.6828 |
| 95% CI |  |  |  |  |  |  |  |  |  |  |
| Lower | 0.7250 | 0.6955 | 0.6882 | 0.8423 | 0.6696 | 0.7425 | 0.6895 | 0.6126 | 0.8268 | 0.6402 |
| Upper | 0.7930 | 0.7657 | 0.7625 | 0.9094 | 0.7486 | 0.8132 | 0.7710 | 0.7102 | 0.9019 | 0.7253 |
| Best threshold | 45.5000 | 23.7950 | 118.5000 | 5.7250 | 1.6250 | 50.5000 | 24.7900 | 127.5000 | 5.6350 | 1.3950 |
| Specificity, % | 64.60 | 54.68 | 50.21 | 90.61 | 71.08 | 74.02 | 66.27 | 70.97 | 88.23 | 62.07 |
| Sensitivity, % | 77.42 | 80.65 | 83.23 | 74.19 | 60.65 | 71.63 | 70.21 | 56.03 | 74.47 | 68.09 |
| Accuracy, % | 64.72 | 54.93 | 50.53 | 90.46 | 70.98 | 73.99 | 66.30 | 70.84 | 88.11 | 62.13 |
| PPV, % | 2.07 | 1.69 | 1.59 | 7.09 | 1.98 | 2.38 | 1.81 | 1.68 | 5.30 | 1.56 |
| NPV, % | 99.66 | 99.66 | 99.68 | 99.73 | 99.47 | 99.66 | 99.60 | 99.46 | 99.74 | 99.55 |
| PLR | 2.1869 | 1.7795 | 1.6716 | 7.9035 | 2.0969 | 2.7567 | 2.0815 | 1.9300 | 6.3254 | 1.7952 |
| NLR | 0.3496 | 0.3540 | 0.3341 | 0.2848 | 0.5537 | 0.3833 | 0.4495 | 0.6196 | 0.2894 | 0.5141 |
| DOR | 6.2561 | 5.0275 | 5.0037 | 27.7510 | 3.7871 | 7.1924 | 4.6306 | 3.1149 | 21.8579 | 3.4916 |

AUC, Area under curve; CI, Confidence interval; PPV, Positive predictive value; NPV, Negative predictive value; PLR, Positive likelihood ratio; NLR, Negative likelihood ratio; DOR, Diagnostic odds ratio.

**Table S3 Prediction performance of other similar risk prediction models for diabetes in China.**

| Prediction  models | Sample size | Age  (year) | Risk factors | Optimal cut-off value | AUC (95% CI) | Sensitivity  (%) | Specificity  (%) |
| --- | --- | --- | --- | --- | --- | --- | --- |
| XIANGHAI ZHOU et al,2013 [1] | 41,809 | 20–74 | age, gender, WC, BMI,SBP and family history of DM. | 25 | 0.748  (0.739–0.756) | 92.3 | 35.5 |
| K. Chien et al,2009 [2] | 2,960 | ≥35 | age, BMI, FPG, TG, HDL-C，and white blood cell count | 13 | 0.702 (0.676–0.727) | 52 | 78 |
| Kun Wang et al, 2019 [3] | 5,557 | -- | age, BMI, FPG, TG, HDL-C, LDL-C, | Female: −2.45  Male: −1.94 | Female: 0.847 (0.801–0.892)  Male: 0.755  (0.717–0.794) | Female :82.6  Male :73.8 | Female :79.1  Male :65.7 |
| Zhong Xin et al, 2010 [4] | 2,261 | ≥35 | age,WHR, WC, duration of hypertension,  and family history of DM. | -- | 0.731 | 74.6 | 71.6 |
| Min Liu et al, 2011 [5] | 1,851 | 40–90 | age, BMI, hypertension, history of high blood glucose, FPG,TG,HDL-C | 4 | 0.734 (0.702–0.766 | 64.5 | 71.6 |
| Jing Xie et al, 2010 [6] | 15,540 | 35-74 | Female:age WHR  Male:age,WC |  | 0.71  0.65 | 61  59 | 71  63 |
| Carlos K.H. WONG et al, 2016 [7] | 2,518 |  | family history of DM, history of  hypertension, BMI,WC ,SBP, DBP, TG, TC, HDL-C, and LDL-C | Non-laboratory-based  Algorithm:  18  Laboratory-based  Algorithm:18 | Non-laboratory-based  Algorithm:  0.686 (0.650, 0.722) Laboratory-based  Algorithm: 0.696 (0.661, 0.731) | Non-laboratory-based  Algorithm:  :57.9  Laboratory-based  Algorithm 66.2 | Non-laboratory-based  Algorithm:  68.9  Laboratory-based  Algorithm :60.2 |
| Feng Sun et al, 2009 [8] | 73,961 | 35-74 | age，gender, education，Family history of T2DM，current smoking，hypertension，BMI，WC，FPG，TG, HDL-C, ALT, eGFR | 15.04 | 0.853（0.834- 0.872） | 72.88 | 83.40 |
| Senlin Luo et al, 2014 [9] | 16,246 | ≥20 | age, gender, BMI, WC, TC, TG, HDL-C, DBP and prior history of diabetes and parental or sibling history of DM | 2.2 | 0.808 | 79.4 | 67.9 |
| X. Chen et al, 2017 [10] | 28,251 | ≥18 | age, BMI, family history of T2DM, diet,  hypertension, FPG | 7 | 0.754 | 63.1 | 75.9 |
| W. G. Gao et al, 2010 [11] | 6,322 | 20–74 | age, WC and family history of DM | 14 | 0.673(0.649–  0.697) | 84.2 | 39.8 |
| Xingwang Ye et al, 2014 [12] | 2,529 | 50–70 | gender, hypertension, BMI, FPG, HbA1c, and CRP | 7 | 0.728  (0.705-0.751) | 55.5 | 75.4 |

DM, Diabetes mellitus; T2DM, Type 2 diabetes mellitus; WC, Waist circumference; WHR, Waist/hip ratio; BMI, Body mass index ; SBP, Systolic blood pressure; DBP, Diastolic blood pressure; FPG, Fasting plasma glucose; TG, Triglyceride ; TC, Total cholesterol; HDL-C, High-density lipoprotein cholesterol; LDL-C, Low-density lipid cholesterol; ALT, Alanine aminotransferase; eGFR, Estimated glomerular filtration rate; HbA1c, Glycated hemoglobin A1C; CRP, C-reactive protein; AUC, Area under curve; CI, Confidence intervals.

**Table S4 Comparison of** **baseline characteristics between the external validation cohort and the overall population of the original study.**

| Characteristic | External validation cohort | The overall population | Standardized Difference | P value |
| --- | --- | --- | --- | --- |
| Participants | 12545 | 211833 |  |  |
| Age (year) | 43.56 ± 8.68 | 42.10 ± 12.65 | 0.13 | <0.001 |
| Gender |  |  | 1.82 (1.80, 1.84) | <0.001 |
| Male | 5681 (45.28%) | 116123 (54.82%) |  |  |
| Female | 6864 (54.72%) | 95710 (45.18%) |  |  |
| BMI (kg/m2) | 22.11 ± 3.11 | 23.24 ± 3.34 | 0.35 | <0.001 |
| SBP (mmHg) | 114.42 ± 14.89 | 119.06 ± 16.38 | 0.30 | <0.001 |
| DBP (mmHg) | 71.63 ± 10.36 | 74.18 ± 10.81 | 0.24 | <0.001 |
| FPG (mmol/L) | 5.15 ± 0.41 | 4.92 ± 0.61 | 0.45 | <0.001 |
| TG (mmol/L) | 0.75 (0.50-1.12) | 1.07 (0.73-1.62) | 0.50 | <0.001 |
| HDL-C (mmol/L) | 1.44 ± 0.39 | 1.37 ± 0.31 | 0.21 | <0.001 |
| ALT (U/L) | 17.00 (13.00-23.00) | 18.00 (13.00-27.70) | 0.20 | <0.001 |
| Follow-up (year) | 2.93 ± 0.23 | 2.68 ± 0.41 | 0.77 | <0.001 |
| Incident diabetes |  |  | 0.07 (0.05, 0.09) | <0.001 |
| No | 12505 (99.68%) | 210069 (99.17%) |  |  |
| Yes | 40 (0.32%) | 1764 (0.83%) |  |  |

Values are n (%) or mean ± SD

BMI, Body mass index; SBP, Systolic blood pressure; DBP, Diastolic blood pressure; FPG; Fasting plasma glucose; TG, Triglyceride; HDL-C, High density lipoprotein cholesterol; ALT, Alanine aminotransferase.

**Table S5 Prediction performance of the external validation cohort and** **the overall population of the original study for the risk of diabetes.**

|  | AUC | 95%  Lower | CI  Upper | Best  threshold | Specificity  (%) | Sensitivity  (%) | Accuracy  (%) | PPV  (%) | NPV  (%) | PLR | NLR | DOR |
| --- | --- | --- | --- | --- | --- | --- | --- | --- | --- | --- | --- | --- |
| External validation | 0.8488 | 0.8126 | 0.8850 | -4.8801 | 81.46 | 75.25 | 81.42 | 2.60 | 99.80 | 4.0591 | 0.3039 | 13.3587 |
| The overall population | 0.9176 | 0.9106 | 0.9246 | -4.6455 | 86.17 | 83.90 | 86.16 | 4.85 | 99.84 | 6.0683 | 0.1868 | 32.4807 |

AUC, Area under curve; CI, Confidence interval; PPV, Positive predictive value; NPV, Negative predictive value; PLR, Positive likelihood ratio; NLR, Negative likelihood ratio; DOR, Diagnostic odds ratio.

**A**


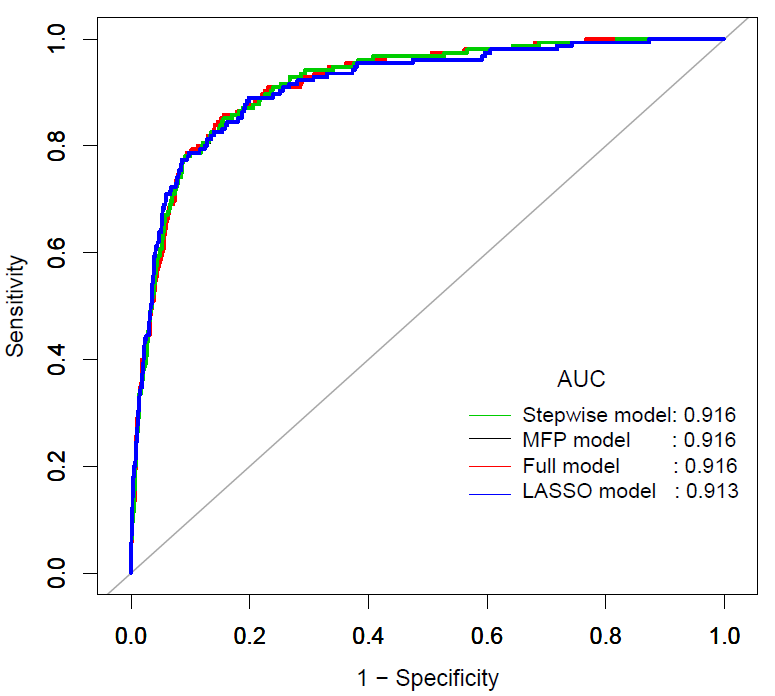


**B**


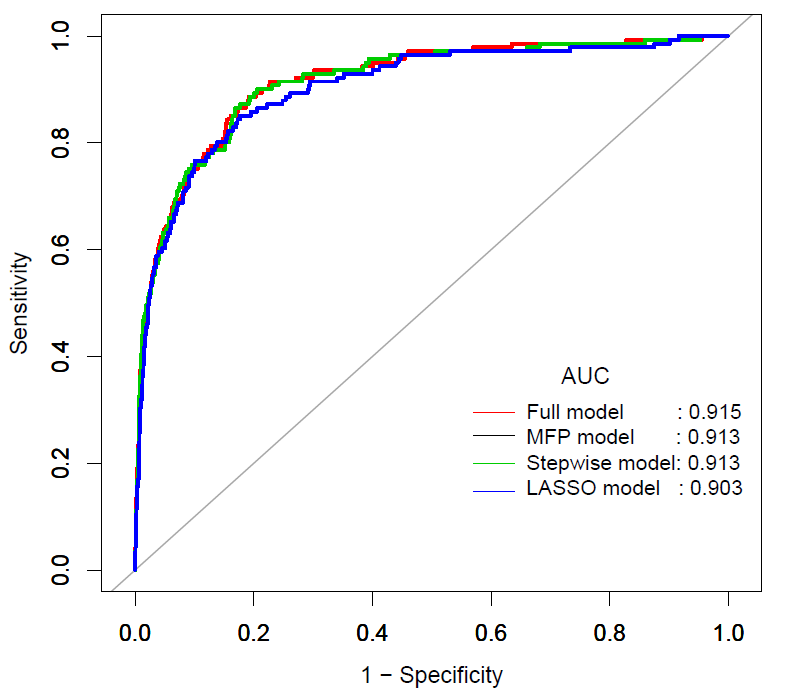


Fig S1. The ROC curves of the LASSO model, full model, stepwise model and MFP model in the training cohort (a) and validation cohort (b).

**A**


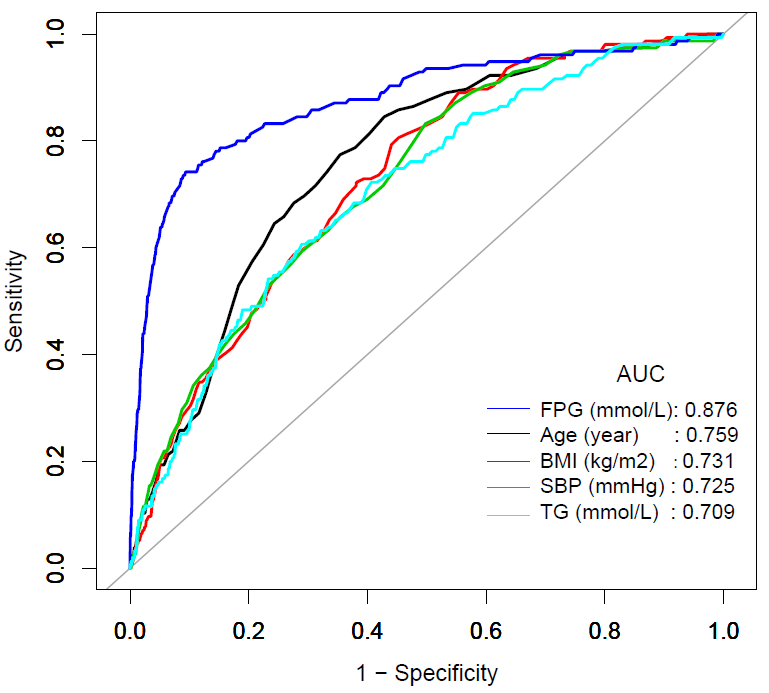


**B**


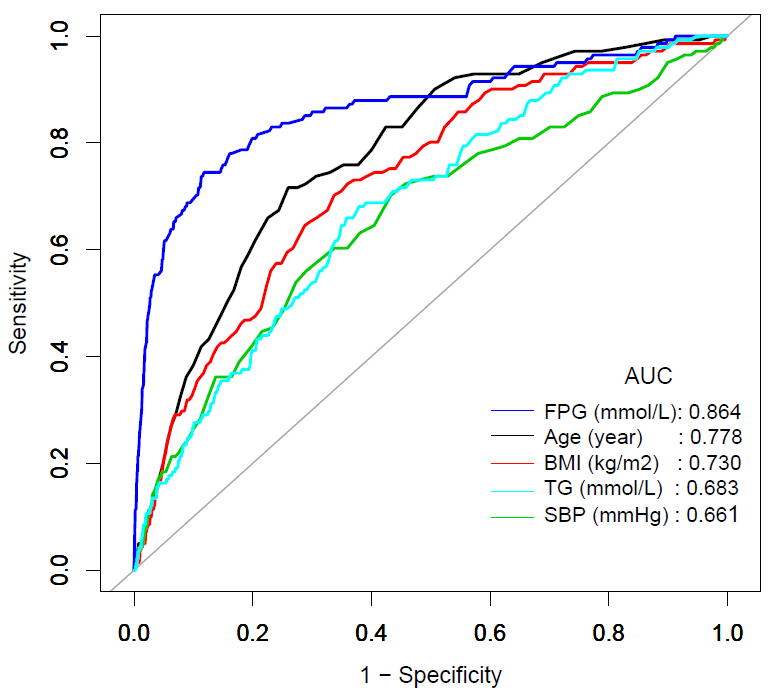


Fig S2. The ROC curves of each risk predictor in our nomogram, including age, BMI, SBP, FPG and TG in the training cohort (a) and validation cohort (b).

**A**


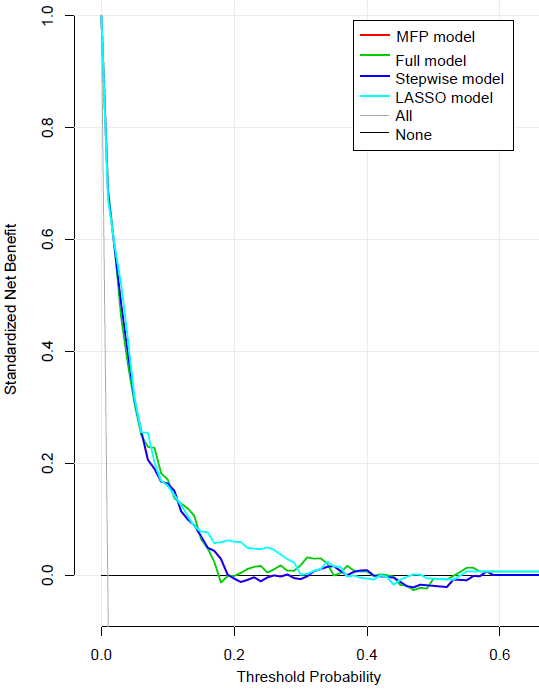


**B**


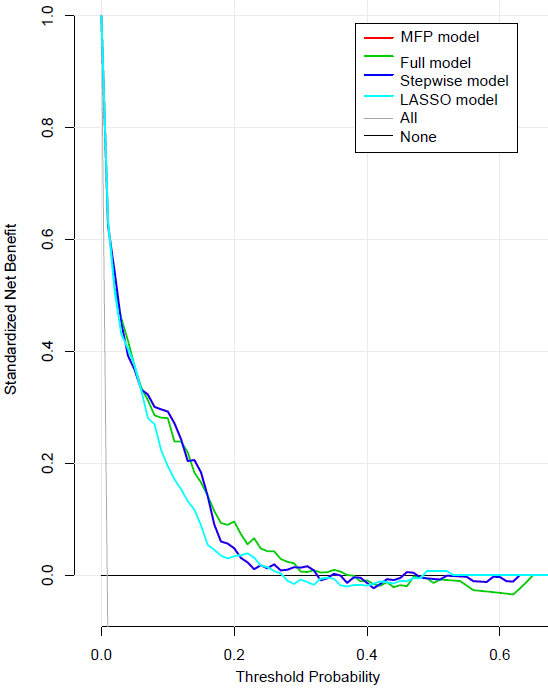


Fig S3. The decision curve analysis of the full model, stepwise model, MFP model and LASSO model for 3-year diabetes risk in the training cohort (a) and validation cohort (b). The black line represents the net benefit when none of the participants are considered to develop diabetes, while the light gray line represents the net benefit when all participants are considered to develop diabetes. The area between the "no treatment line" (black line) and "all treatment line" (light gray line) in the model curve indicates the clinical utility of the model. The farther the model curve is from the black and light gray lines, the better the clinical use of the nomogram. (Using bootstraps with 500 resamples)
